# Functional genetic variant of WW domain-containing oxidoreductase (WWOX) gene is associated with hepatocellular carcinoma risk

**DOI:** 10.1371/journal.pone.0176141

**Published:** 2017-04-20

**Authors:** Hsiang-Lin Lee, Hsin-Lin Cheng, Yu-Fan Liu, Ming-Chih Chou, Shun-Fa Yang, Ying-Erh Chou

**Affiliations:** 1 Institute of Medicine, Chung Shan Medical University, Taichung, Taiwan; 2 School of Medicine, Chung Shan Medical University, Taichung, Taiwan; 3 Deptartment of Surgery, Chung Shan Medical University Hospital, Taichung, Taiwan; 4 Department of Biomedical Sciences, Chung Shan Medical University, Taichung, Taiwan; 5 Department of Medical Research, Chung Shan Medical University Hospital, Taichung, Taiwan; Chang Gung Memorial Hospital Kaohsiung Branch, TAIWAN

## Abstract

**Background:**

Hepatocellular carcinoma (HCC) is one of the most common malignant tumors worldwide. Human WW domain-containing oxidoreductase (*WWOX*) gene has been identified as a tumor suppressor gene in multiple cancers. We hypothesize that genetic variations in *WWOX* are associated with HCC risk.

**Methodology/Principal findings:**

Five single-nucleotide polymorphisms (SNPs) of the *WWOX* gene were evaluated from 708 normal controls and 354 patients with HCC. We identified a significant association between a *WWOX* single nucleotide polymorphism (SNP), rs73569323, and decreased risk of HCC. After adjustment for potential confounders, patients with at least one T allele at rs11545028 of *WWOX* may have a significantly smaller tumor size, reduced levels of α-fetoprotein and alanine aminotransferase (ALT). Moreover, the A allele at SNP rs12918952 in *WWOX* conferred higher risk of vascular invasion. Additional in silico analysis also suggests that *WWOX* rs12918952 polymorphism tends to affect WWOX expression, which in turn contributes to tumor vascular invasion.

**Conclusions:**

In conclusion, genetic variations in *WWOX* may be a significant predictor of early HCC occurrence and a reliable biomarker for disease progression.

## Introduction

Primary liver cancer, particularly hepatocellular carcinoma (HCC), has emerged as the third leading cause of cancer-related death worldwide[1] and has been ranked as the second most prevalent malignant cancer in Taiwan. Currently, hepatic resection and liver transplantation are widely recognized as effective therapeutic options for HCC [2, 3]. However, the tumor recurrence rate at 5 years after hepatic resection is approximately 70% [4, 5]. Etiologically, HCC is a complex malignancy that has been associated with various risk factors, including chronic hepatitis B (HBV) infection, excessive alcohol intake, and metabolic diseases [6]. In addition to known etiologies, studies have also suggested that the impact of genetic factors within the coding and noncoding regions of tumor suppressor genes decreases gene expression and increases the carcinogenesis of HCC or intrahepatic metastasis of the primary tumor [7–9].

The human WW domain-containing oxidoreductase (*WWOX*) gene, located on chromosome 16q23.3–24.1, spans one of the most active fragile sites, which contains the FRA16D. *WWOX* is a *bona fide* tumor suppressor gene, which plays a pivotal role in regulating signaling pathways and cellular functions [10–16]. Studies have suggested that WWOX can induce apoptosis both in vivo and in vitro by interacting with p53, p73, and JNK1 [11, 17]. Moreover, Hsu et al. concluded that TGF-β1 and hyaluronan can activate HYAL-2-WWOX-SMAD4 signaling to cause cell death [15]. Additionally, evidence from in vivo overexpression studies suggests that WWOX might suppress the carcinogenetic effect of MDA-MB-435 breast cancer cells [18]. However, low or decreased expression and aberrant transcription of *WWOX* has been reported in several types of cancer, including nonsmall cell lung cancer (85%), prostate cancer (84%), breast cancer (63%), and oral cancer (40%) [19–24]. Moreover, Aqeilan et al. created mice carrying a targeted deletion of the WWOX gene to observe incidence of tumor formation. The results shown that WWOX is a bona fide tumor suppressor [25]. In addition, the loss of WWOX expression is correlated with higher tumor stages and less favorable outcomes in patients [26].

Recently, the genetic alteration of the *WWOX* gene showed a high incidence of loss of expression in squamous cell lung carcinoma [27, 28]. Notably, single nucleotide polymorphisms (SNPs) in various genes reportedly modulate their expression and are associated with the risk of several cancers [29, 30]. In addition, SNPs within the WWOX gene have been identified as a potentially genetic risk factor for esophageal adenocarcinoma and multiple myeloma [31, 32]. Recently, genome-wide linkage analysis studies for SNPs in prostate cancer patients have revealed that WWOX polymorphic variants may be associated with cancer susceptibility [33]. Of these, the *WWOX* polymorphism is considered to be a useful and tractable measure to evaluate the associations between the SNPs and clinicopathological characteristics of cancers.

Considering the potential function of the *WWOX* gene in the neoplastic process, SNPs in this gene may be associated with HCC risk. To test this hypothesis, we performed a hospital-based study to evaluate the impact of gene variations of *WWOX* on the development of HCC and observed an association of a *WWOX* SNP (rs12918952) with the risk and progression of HCC.

## Materials and methods

### Patient specimens

In 2007–2015, for the case group, we recruited 354 patients (252 men and 102 women; mean age = 62.97 ± 11.60 years; age range = 30–90 years) at Chung Shan Medical University Hospital in Taichung, Taiwan. During the same study period, the 708 individuals (504 men and 204 women) were enrolled as these subjects received a physical examination at the same hospital. Patients with only HCC were recruited. Patients and normal controls were excluded if having any history of other cancers. For both groups, we administered a questionnaire to obtain information on their exposure to tobacco use, and alcohol consumption. Medical information of the patients, including TNM clinical staging, primary tumor size, and lymph node involvement was obtained from their medical records. Before commencing the study, approval was obtained from the Institutional Review Board of Chung Shan Medical University Hospital, and informed written consent was obtained from each individual.

### Sample preparation and DNA extraction

The whole blood specimens, collected from controls and HCC patients, were placed in tubes containing EDTA and were immediately centrifuged. The genomic DNA was extracted from buffy coats using a QIAamp DNA blood mini kits as described in detail previously [34]. DNA was dissolved in TE buffer and used as the template in polymerase chain reactions.

### SNP selection and genotyping

In this study, the selection of 5 well-characterized common polymorphisms from *WWOX* gene is based on their wide associations with the development of cancer [35, 36]. We included rs11545028 in the 5’UTR region. Rs12918952 and rs3764340, which are located in the exon of WWOX, were selected in this study since these 2 SNPs may result from amino acid changes and thus the loss of the tumor suppression function of WWOX [37]. The allelic discrimination of *WWOX* rs11545028 (Assay ID: C_2813530_10), rs12918952 (Assay ID: C_57888_20), rs3764340 (Assay ID: C_25654217_20), rs73569323 (Assay ID: C_25761998_10), and rs383362 (Assay ID: C_2395473_20) polymorphisms were assessed using an ABI StepOne TM Real-Time PCR System (Applied Biosystems, Foster City, CA) and analyzed using SDS v3.0 software (Applied Biosystems, Foster City, CA).

### Bioinformatics analysis

Several semi-automated bioinformatics tools were applied to assess whether *WWOX* rs12918952 SNPs are associated with a putative function that might affect patient outcomes. GTEx database[38] from the ENCODE project [39] were used to identify the regulatory potential of candidate functional variants. The GTEx data were used to identify correlations between SNPs and whole blood-specific gene expression levels.

### Statistical analysis

Mann–Whitney U-test and Fisher’s exact test were used to compare the age, gender differences and demographic characteristic distributions between the controls and patients with HCC. The odds ratio and 95% CIs of the association between the genotype frequencies and HCC risk and the clinical pathological characteristics were estimated using multiple logistic regression models. *p* < 0.05 was considered significant. The data were analyzed on SAS statistical software (Version 9.1, 2005; SAS Institute, Cary, NC).

## Results

### Characteristics of study participants

The demographic characteristics and clinical parameters of the 2 study groups, including age, sex, and alcohol and tobacco consumption, are shown in Table 1. A significant difference in the alcohol consumption (p < 0.001) was observed between healthy controls and HCC patients, whereas no such significant between-group difference was observed in the tobacco consumption (p = 0.350). However, neither age (p = 0.287) nor sex (p = 1.000) elevated the HCC risk.

**Table 1:** The distributions of demographical characteristics and clinical parameters in 708 controls and 354 patients with HCC.

Variable	Controls (N = 708)	Patients (N = 354)	p value
**Age (yrs)**			
≤60	274 (38.7%)	149 (42.1%)	p = 0.287
>60	434 (61.3%)	205 (57.9%)	
**Gender**			
Male	504 (71.2%)	252 (71.2%)	p = 1.000
Female	204 (28.8%)	102 (28.8%)	
**Alcohol consumption**			
No	597 (84.3%)	224 (63.3%)	p<0.001
Yes	111 (15.7%)	130 (36.7%)	
**Tobacco consumption**			
No	441 (62.3%)	210 (59.3%)	p = 0.350
Yes	267 (37.7%)	144 (40.7%)	
**Stage**			
I+II		233 (65.8%)	
III+IV		121 (34.2%)	
**Tumor T status**			
≤T2		237 (66.9%)	
>T2		117 (33.1%)	
**Lymph node status**			
N0		342 (96.6%)	
N1+N2		12 (3.4%)	
**Metastasis**			
M0		336 (94.9%)	
M1		18 (5.1%)	
**vascular invasion**			
No		292 (82.5%)	
Yes		62 (17.5%)	

### Association between WWOX gene polymorphisms and HCC

Table 2 shows the genotype distributions and associations between HCC patients and healthy controls with WWOX polymorphisms. The alleles with the highest distribution frequency at WWOX rs11545028, rs12918952, rs3764340, rs73569323, and rs383362 in HCC patients and controls were homozygous for C/C, homozygous for G/G, homozygous for C/C, homozygous for C/C, and homozygous for G/G, respectively. After adjustment for several variables, individuals with polymorphisms at rs11545028, rs12918952, rs3764340, and rs383362 showed no reduction in the risk of HCC. However, compared with the wild-type individuals, individuals carrying C/T or C/T+T/T at rs73569323 exhibited a 0.305-fold (95% CI: 0.126–0.741) or 0.299-fold (95% CI: 0.123–0.724; both p <0.05) lower risk of HCC, respectively.

**Table 2:** Distribution frequency of *WWOX* genotypes in 708 controls and 354 patients with HCC.

Variable	Controls (N = 708) n (%)	Patients (N = 354) n (%)	OR (95% CI)	AOR (95% CI)
**rs11545028**				
CC	410 (57.9%)	212 (59.9%)	1.00	1.00
CT	261 (36.9%)	124 (35.0%)	0.919 (0.701–1.204)	0.950 (0.719–1.255)
TT	37 (5.2%)	18 (5.1%)	0.941 (0.523–1.692)	0.939 (0.513–1.719)
CT+TT	298 (42.1%)	142 (40.1%)	0.922 (0.711–1.195)	0.949 (0.726–1.239)
**rs12918952**				
GG	637 (90.0%)	310 (87.6%)	1.00	1.00
GA	70 (9.9%)	42 (11.9%)	1.233 (0.822–1.850)	1.179 (0.775–1.793)
AA	1 (0.1%)	2 (0.5%)	4.110 (0.371–45.496)	3.933 (0.333–46.441)
GA+AA	71 (10.0%)	44 (12.4%)	1.273 (0.854–1.899)	1.218 (0.806–1.840)
**rs3764340**				
CC	594 (83.9%)	290 (81.9%)	1.00	1.00
CG	106 (15.0%)	63 (17.8%)	1.217 (0.865–1.714)	1.236 (0.869–1.757)
GG	8 (1.1%)	1 (0.3%)	0.256 (0.032–2.057)	0.241 (0.029–2.007)
CG+GG	114 (16.1%)	64 (18.1%)	1.150 (0.821–1.610)	1.163 (0.823–1.646)
**rs73569323**				
CC	669 (94.5%)	348 (98.3%)	1.00	1.00
CT	38 (5.4%)	6 (1.7%)	0.304 (0.127–0.725)*	0.305 (0.126–0.741)*
TT	1 (0.1%)	0 (0%)	----	----
CT+TT	39 (5.5%)	6 (1.7%)	0.296 (0.124–0.705)*	0.299 (0.123–0.724)*
**rs383362**				
GG	529 (74.7%)	282 (79.7%)	1.00	1.00
GT	170 (24.0%)	70 (19.8%)	0.772 (0.564–1.057)	0.744 (0.538–1.028)
TT	9 (1.3%)	2 (0.6%)	0.265 (0.089–1.943)	0.481 (0.101–2.290)
GT+TT	179 (25.3%)	72 (20.3%)	0.755 (0.554–1.028)	0.732 (0.532–1.006)

The odds ratios (ORs) and with their 95% confidence intervals (CIs) were estimated by logistic regression models. The adjusted odds ratios (AORs) with their 95% confidence intervals (CIs) were estimated by multiple logistic regression models after controlling for alcohol consumption.

* p value < 0.05 as statistically significant.

### Correlation between polymorphic genotypes of WWOX and clinical status of HCC

The distributions of the clinicopathological characteristics and WWOX genotypes in HCC patients were further explored (Tables 3 and 4). As shown in Table 3, patients with at least one polymorphic allele of rs11545028 (C/T or T/T genotype) were less prone to develop large tumors (p = 0.039). In addition, we observed that the trend of G/A+G/G genotype of WWOX rs12918952 for vascular invasion risk (p = 0.024) was higher in male patients with HCC. Furthermore, we also examined the potential association between WWOX gene polymorphisms and the clinicopathological markers of HCC, including α-fetoprotein, aspartate aminotransferase (AST), alanine aminotransferase (ALT), and the AST/ALT ratio. However, we observed significantly lower α-fetoprotein and ALT levels in patients who carried the rs11545028 C/T or T/T genotypes (p = 0.013 and 0.041, respectively; Table 5). Furthermore, in HCC patients, compared with the C/C genotype, the C/G and G/G genotype of WWOX rs3764340 were associated with higher AST and ALT levels (both p < 0.05).

**Table 3:** Odds ratio (OR) and 95% confidence interval (CI) of clinical status and *WWOX* rs11545028 genotypic frequencies in 354 HCC patients.

Variable	Genotypic frequencies			
	CC (N = 212)	CT+TT (N = 142)	OR (95% CI)	p value
**Clinical Stage**				
Stage I/II	132 (62.3%)	101 (71.1%)	1.00	p = 0.085
Stage III/IV	80 (37.7%)	41 (28.9%)	0.670 (0.424–1.058)	
**Tumor size**				
≦ T2	133 (62.7%)	104 (73.2%)	1.00	p = 0.039*
> T2	79 (37.3%)	38 (26.8%)	0.615 (0.387–0.979)	
**Lymph node metastasis**				
No	205 (96.7%)	137 (96.5%)	1.00	p = 0.911
Yes	7 (3.3%)	5 (3.5%)	1.069 (0.332–3.436)	
**Distant metastasis**				
No	202 (95.3%)	134 (94.4%)	1.00	p = 0.700
Yes	10 (4.7%)	8 (5.6%)	1.206 (0.464–3.134)	
**Vascular invasion**				
No	175 (82.5%)	117 (82.4%)	1.00	p = 0.970
Yes	37 (17.5%)	25 (17.6%)	1.011 (0.578–1.767)	
**Child-Pugh grade**				
A	163 (76.9%)	107 (75.4%)	1.00	p = 0.739
B or C	49 (23.1%)	35 (24.6%)	1.088 (0.662–1.790)	
**HBsAg**				
Negative	123 (58.0%)	87 (61.3%)	1.00	p = 0.542
Positive	89 (42.0%)	55 (38.7%)	0.874 (0.566–1.349)	
**Anti-HCV**				
Negative	114 (53.8%)	66 (46.5%)	1.00	p = 0.178
Positive	98 (46.2%)	76 (53.5%)	1.340 (0.875–2.051)	
**Liver cirrhosis**				
Negative	41 (19.3%)	29 (20.4%)	1.00	p = 0.802
Positive	171 (80.7%)	113 (79.6%)	0.934 (0.549–1.590)	

The ORs with analyzed by their 95% CIs were estimated by logistic regression models.

> T2: multiple tumor more than 5 cm or tumor involving a major branch of the portal or hepatic vein(s)

* p value < 0.05 as statistically significant.

**Table 4:** Odds ratio (OR) and 95% confidence interval (CI) of clinical status and *WWOX* rs12918952 genotypic frequencies in 252 male HCC patients.

Variable	Genotypic frequencies			
	GG (N = 224)	GA+AA (N = 28)	OR (95% CI)	p value
**Clinical Stage**				
Stage I/II	139 (62.1%)	22 (78.6%)	1.00	p = 0.086
Stage III/IV	85 (37.9%)	6 (21.4%)	0.446 (0.174–1.144)	
**Tumor size**				
≦ T2	140 (62.5%)	22 (78.6%)	1.00	p = 0.094
> T2	84 (37.5%)	6 (21.4%)	0.455 (0.177–1.166)	
**Lymph node metastasis**				
No	115 (96.0%)	27 (96.4%)	1.00	p = 0.909
Yes	9 (4.0%)	1 (3.6%)	0.885 (0.108–7.257)	
**Distant metastasis**				
No	211 (94.2%)	27 (96.4%)	1.00	p = 0.627
Yes	13 (5.8%)	1 (3.6%)	0.601 (0.076–4.778)	
**Vascular invasion**				
No	190 (84.8%)	19 (67.9%)	1.00	p = 0.024*
Yes	34 (15.2%)	9 (32.1%)	2.647 (1.106–6.338)	
**Child-Pugh grade**				
A	174 (77.7%)	21 (75.0%)	1.00	p = 0.749
B or C	50 (22.3%)	7 (25.0%)	1.160 (0.466–2.886)	
**HBsAg**				
Negative	120 (53.6%)	16 (57.1%)	1.00	p = 0.721
Positive	104 (46.4%)	12 (42.9%)	0.865 (0.391–1.913)	
**Anti-HCV**				
Negative	127 (56.7%)	15 (53.6%)	1.00	p = 0.753
Positive	97 (43.3%)	13 (46.4%)	1.135 (0.516–2.496)	
**Liver cirrhosis**				
Negative	47 (21.0%)	4 (14.3%)	1.00	p = 0.406
Positive	177 (79.0%)	24 (85.7%)	1.593 (0.527–4.816)	

The ORs with analyzed by their 95% CIs were estimated by logistic regression models.

> T2: multiple tumor more than 5 cm or tumor involving a major branch of the portal or hepatic vein(s)

* p value < 0.05 as statistically significant.

**Table 5:** Association of *WWOX* genotypic frequencies with HCC laboratory status.

Characteristic	α-Fetoprotein ^a^ (ng/mL)	AST ^a^(IU/L)	ALT ^a^(IU/L)	AST/ALT ratio ^a^
**rs11545028**				
CC	5119.4 ± 1411.9	156.8 ± 23.3	134.4 ± 18.4	1.55 ± 0.12
CT+TT	799.5 ± 229.9	101.1 ± 14.4	84.6 ± 11.1	1.36 ± 0.07
p value	0.013*	0.072	0.041*	0.232
**rs12918952**				
GG	3785.0 ± 975.8	136.5 ± 16.9	116.5 ± 13.5	1.46 ± 0.08
GA+AA	579.4 ± 345.3	120.3 ± 25.3	99.6 ± 15.2	1.54 ± 0.23
p value	0.218	0.725	0.642	0.729
**rs3764340**				
CC	3655.0 ± 991.4	99.9 ± 8.5	93.9 ± 8.5	1.40 ± 0.06
CG+GG	2170.3 ± 1520.9	291.0 ± 71.7	290.96 ± 71.7	1.78 ± 0.31
p value	0.506	<0.001*	<0.001*	0.059
**rs73569323**				
CC	3443.6 ± 871.7	135.5 ± 15.4	115.1 ± 12.1	1.48 ± 0.08
CT+TT	78.1 ± 37.1	70.7 ± 25.6	72.7 ± 29.1	1.08 ± 0.11
p value	0.613	0.581	0.647	0.507
**rs383362**				
GG	3317.0 ± 931.6	138.8 ± 18.6	116.8 ± 14.5	1.53 ± 0.10
GT+TT	3659.3 ± 2122.9	117.3 ± 16.2	105.1 ± 15.0	1.24 ± 0.06
p value	0.873	0.569	0.695	0.124

Mann-Whitney U test was used between two groups.

^a^ Mean ± S.E.

* p value < 0.05 as statistically significant.

### Functional analysis of the WWOX rs12918952 locus

**Fig 1:**
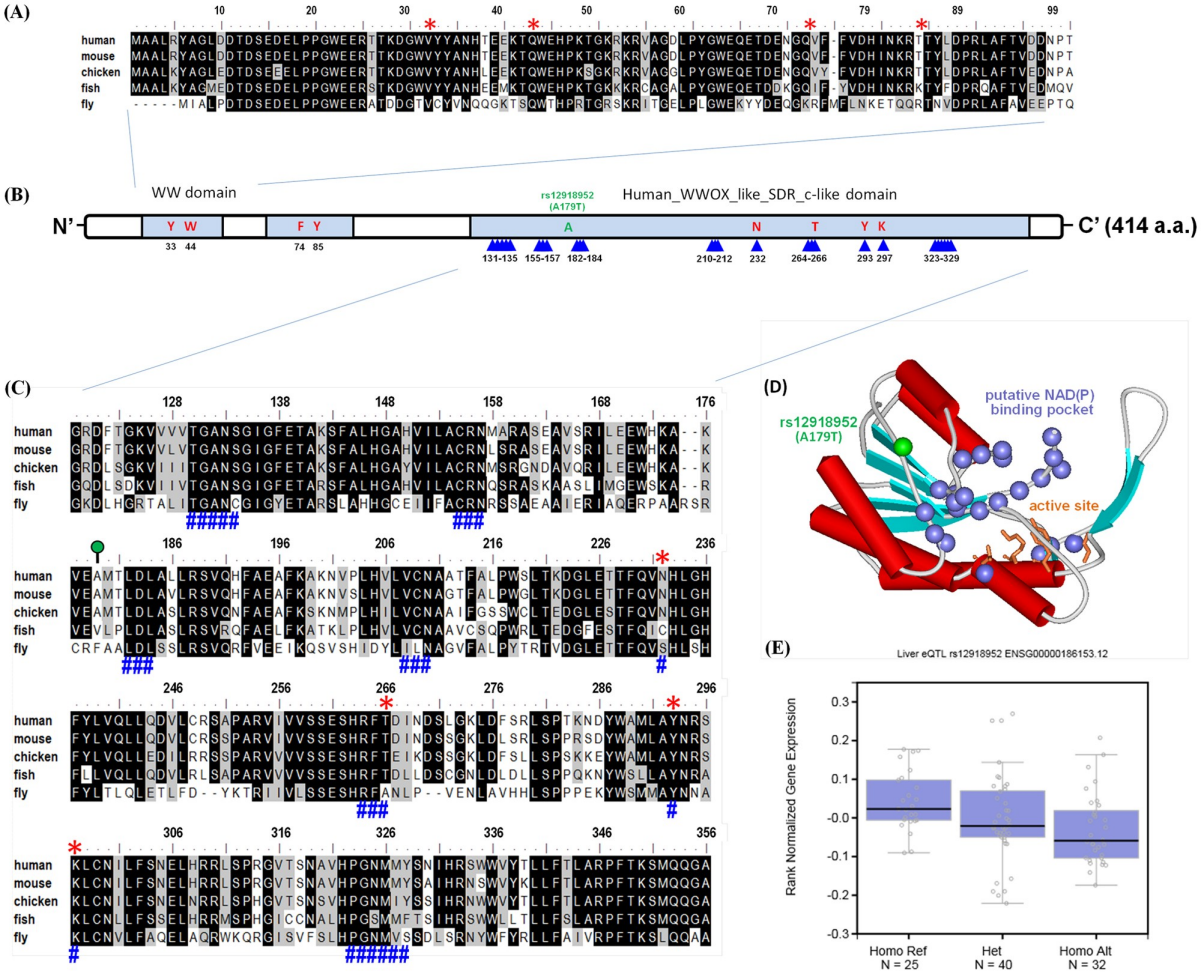
Structural characterization and SNP (rs12918952) in human WWOX protein (NP_057457.1). Alignments of conserved domain-based sequences of (A) two conserved tryptophans domain (WW; cd00201) and (C) classical-like SDR domain (human_WWOX-like_SDR_c-like; cd09809) by use of multiple sequence alignment format and numbered according to the human WWOX is shown above the sequences. Strick consensus amino acids in the putative active centers of compact structural units are shown: the key amino acids of active site and cofactor binding site are highlighted in red star and blue pound signs, respectively. The WWOX-relative sequences are as follows: human (*H*. *sapiens*, NP_057457.1); mouse (*M*. *musculus*, NP_062519.2); chicken (*G*. *gallus*, NP_001025745.1); fish (*D*. *rerio*, NP_957207.1) and fly (*D*. *melanogaster*, NP_609171.1). (B) Schematic representation of the overall human WWOX protein; domain symbols are drawn approximately to scale. The rectangle represents the WW and human_WWOX-like_SDR_c-like domain; the key residues of active site and cofactor binding site are highlighted in red and triangle sign, respectively. The N-terminal and C-terminal ends are indicated (N’ and C’, respectively). (D) Ribbon diagram showing the homologous 3D model of the SDR_c-like domain of human WWOX using the SWISSMODEL server based on *M*. *abscessus* short chain dehydrogenase or reductase crystal structure (PDB ID: 3RIH). The side chains of the amino acids characterized catalytic tetrad of Asn232-Thr266-Tyr293-Lys297 are shown as sticks and labeled which aligned well with the ‘classical’ type of short-chain dehydrogenase/reductase (SDR) enzyme. The blue spheres represent the putative coenzyme NAD (P)-binding pocket is drawn to illustrate the location of the cofactor binding site. The ribbons indicate the backbone course of human WWOX and the arrows represent b-strands, and cylinders indicate a-helices. The figure was prepared using ViewerLite^™^ 5.0 software. (E) Expression quantitative trait locus association between rs12918952 genotype and WWOX expression in whole blood (GTEx data set). Numbers in parentheses indicate the number of cases.

To validate whether the Ala-179-Thr substitution influenced gene expression levels, we used multiple sequence alignment and 3-dimensional (3D) structures to understand the locations of amino acid residues and protein structures for the preliminary assessment of the putative functional roles of SNPs. Multiple alignment data showed the portion of the amino acid sequence of the WW domain within *WWOX*, and the Ala-179-Thr variation, which was located near the key amino acid of the cofactor binding site in human *WWOX* like SDR c-like domain (Fig 1A–1C). The homology-based 3D protein structure of WWOX shows the putative coenzyme NAD (P)-binding pocket and predicts the active site using the SWISS-MODEL base on the *M*. *abscessus* short chain dehydrogenase or the reductase crystal structure as a template. The rs12918952 variants are positioned within the functional cofactor binding of the *WWOX* gene (Fig 1D). In addition, given the association of WWOX genetic substitution with gene expression, we used the Genotype–Tissue Expression (GTEx) database to explore whether rs12918952 was associated with the expression of WWOX in human HCC. Individuals carrying rs121918952-variant genotypes (GA or AA) showed a trend of decreased expression of WWOX compared with that of the wild-type homozygous GG genotype (Fig 1E). The rs12918952 G to A substitution probably affects catalytic activity, decreases *WWOX* mRNA expression, and subsequently enhances the vascular invasion of HCC.

## Discussion

Several studies have suggested that *WWOX* polymorphic variants are consistently associated with more aggressive phenotypes and poor outcomes in numerous malignant diseases, including esophageal squamous cell carcinoma, thyroid carcinoma, pancreatic cancer, and lung cancer [36, 40–42]. In the current study, we evaluated variations in the *WWOX* gene and the clinicopathological development of HCC across 2 independent individuals. Furthermore, we reported the additional finding that WWOX expression was downregulated in HCC, which strengthened the evidence of a link between polymorphic variations and susceptibility to HCC.

Alcohol consumption, HBV or HCV infection, history of liver cirrhosis, and family history of HCC are the major etiologic factors for HCC in Taiwan [43, 44]. Our data show that the number of individuals with a history of alcohol consumption was higher among the HCC patient group (36.7%) than among the control participants (15.7%), indicating that alcohol consumption is highly associated with increased HCC risk. Alcohol abuse is known to be carcinogenic in humans and causes oxidative stress in hepatic cells, which play a pivotal role in the etiology of liver damage [45]. Chronic alcohol abuse accelerates hepatobiliary tumors by upregulating miR-122-mediated HIF-1α activity and stemness [46]. Interestingly, moderate alcohol intake altered autophagy- and apoptosis-signaling networks in a pig model [47]. Indeed, exposure to such carcinogens frequently altered genes at fragile sites, which led to the loss of WWOX suppressor function. Consistent with our data, patients with alcohol consumption had a higher risk of developing HCC. Moreover, in the current study, our result shown that SNP rs12918952 in *WWOX* conferred higher risk of vascular invasion, however, no difference was found regarding the HBsAg and anti-hepatitis C virus (Table 4). WWOX is known to regulate virus-associated immunodeficiency and various cancers, including HCC [9, 48–51]. Further investigation is warranted to explore the potential role of *WWOX* polymorphism and viral regulation of HCC progression.

WWOX, a protein bearing the WW domain, interacts with several proteins and plays a principle role in preventing tumorigenesis. Decreased expression or genetic alteration of *WWOX* has been detected in various malignant tumors. The influence of WWOX expression on the regulation of carcinogenesis, cell cycle, and apoptosis has also been recently reported [52–54]. However, these initial associations were not consistent with our expectation that the genetic variants rs73569323 (C1442T) and rs11545028 (C121T) would be significantly associated with a lower risk of HCC and tumor size. Furthermore, the 2 SNPs, C121T in the Kozak translation initiation site and C1442T in a closely micro-RNA target region in 3'-UTR, did not alter critical residues of the WW or SDR domain. Nevertheless, we have previously observed that C121T represents an oncogenic target for the translational dysregulation of *WWOX* expression in OSCC [55]. The clinicopathological implications of micro-RNA targeting the 3'-UTR region and the regulation of gene expression are well determined. Previously, genome-wide studies have investigated the link among *WWOX* genetic variations, such as SNPs and diseases, and have identified a cis-regulatory variation in the intron of *WWOX* [56, 57]. Our results suggest that the polymorphic variants C121T and C1442T seen in HCC are driven by potential enhancer elements within the noncoding regions rather than the variants in the protein-coding regions; this finding indicates that natural variants may be the key primary contributors to WWOX protein expression.

The aforementioned abnormality of WWOX expression contributes to HCC tumorigenesis, which may be associated with all-cause mortality, particularly in people with high AST and ALT levels [7, 58]. Moreover, serum α-fetoprotein level was found to be a pathological biomarker of inflammation and fibrosis in chronic hepatitis B patients [59]. With regard to the clinical status, our results showed that compared with those who carried the CC genotype, the rs11545028 of patients who carried the CT or TT genotype was significantly correlated with low α-fetoprotein and ALT levels; this finding may be related to the cis-enhancing effect within the 5'-flanking region. However, α-fetoprotein levels are an independent predictor for the severity of inflammation and prognosis of HCC, even in chronic hepatitis B patients whose normal or low α-fetoprotein levels still indicated a severe condition [59, 60]. By contrast, the serum ASL and ALT levels in polymorphic rs3764340 are significantly higher in the CG+GG composition with CC. The rs3764340 SNP has been predicted to cause an alteration of the protein structure at codon 282 of WWOX, where an α-helix is disrupted, and this is associated with an elevated risk of developing multiple neoplasias, thereby reflecting the contribution of rs3764340 C>G substitution to high AST and ALT levels [35, 41, 42, 61]. According to these criteria, α-fetoprotein, AST, and ALT are promising biomarkers for the diagnosis of HCC on the basis of the genetic variations of *WWOX* rs11545028 and rs3764340.

In addition, we identified one synonymous variant, rs12918952, in exon 6, which was significantly associated with the vascular invasion of HCC. Further exploration using the GTEx dataset for liver tissue and expression quantitative trait loci analysis revealed that the rs12918952 A allele was associated with decreased WWOX expression. A clinical study of 101 primary bladder tumor samples demonstrated that the low expression of WWOX was correlated with advanced cancer stages and tumor progression [62]. A similar proportion of WWOX downregulation associated with aggressive phenotypes and poor prognosis has been observed in many cancers. Intriguingly, we showed that rs12918952 was mapped in the coding region near the typical coenzyme binding site within the SDR domain. Single-nucleotide substitution in coding or regulatory sequences affects the protein structure, and conformation has been demonstrated. To the best of our knowledge, no previous evidences have revealed that rs12918952 G>A substitution has the potential to modify the biological activity and protein stability of WWOX. Recently, the deletion of *WWOX* exon 6–8 was identified in lung cancer, resulting in a loss of the tumor suppressor function [63]. In the present study, the missense polymorphism rs12918952 G>A located in exon 6 conferred a decreased expression of WWOX, which is partially responsible for the higher vascular invasion of HCC. However, the direct testing of this hypothesis beyond the purpose of the current study is difficult. Nevertheless, it is a unique feature and warrants further investigation.

In conclusion, the present study reports a potential clinically significant finding that several variants of *WWOX* are associated with the clinical status and susceptibility of HCC. Although the clinical and pathological features differ among the various polymorphisms of *WWOX*, our findings still provide a deeper insight into the genetic variations. Additional bioinformative analyses should be conducted to evaluate the clinical utility of predicting the likelihood of patient susceptibility to HCC. Comprehensive variant detection and analysis are prerequisites for developing optimal therapeutic approaches that can eventually ameliorate the clinical phenotype in patients harboring the corresponding lesions.
